# Policy to cover perinatal care costs: a quasi-experimental study on adverse newborn health outcomes

**DOI:** 10.1093/eurpub/ckac129.277

**Published:** 2022-10-25

**Authors:** AM Epure, E Courtin, P Wanner, A Chiolero, S Cullati

**Affiliations:** 1 Population Health Laboratory, University of Fribourg, Fribourg, Switzerland; 2 Department of Public Health, Environments and Society, LSHTM, London, UK; 3 Institute of Demography and Socioeconomics, University of Geneva, Geneva, Switzerland; 4 Institute of Primary Health Care, University of Bern, Bern, Switzerland; 5 School of Population and Global Health, McGill University, Montreal, Canada; 6 Department of Readaptation and Geriatrics, University of Geneva, Geneva, Switzerland

## Abstract

**Background:**

Low birth weight (LBW) and preterm birth are associated with an increased risk of neonatal death and chronic conditions across the life course. Reducing LBW is a global public health priority and requires strategies to improve healthcare during pregnancy. We aimed to assess the effect of a health policy providing full coverage of illness-related costs from 13 weeks of gestation through 8 weeks postpartum on birth outcomes and neonatal mortality in Switzerland.

**Methods:**

We applied a regression discontinuity design to administrative data gathered as part of a Swiss research program (NCCR on the Move). We included all children (N = 166,709) born between March 1, 2013 and February 28, 2015. The outcomes were birth weight (BW), gestational age (GA), LBW (<2,500 g) and very low birth weight (VLBW; <1,500 g), preterm (<37 weeks of gestation), and extremely preterm (<28 weeks), and neonatal (≤ 28 days) death. Children were exposed to the policy if they were born from March 1, 2014 onwards. We estimated the intention-to-treat effect of the policy using parametric regression models.

**Results:**

Children had a mean BW of 3,291 g and mean GA of 275 days. The prevalence of LBW was 6.4%, VLBW 1%, preterm 7.2%, and extremely preterm 0.4%, respectively. Some 0.3% newborn died within one month. The policy increased BW (mean difference =13 g [95% confidence interval (CI): 1, 25]) and decreased the risk of LBW (odds ratio [OR]=0.89; 95% CI: 0.82, 0.98) and VLBW (OR = 0.81; 95% CI: 0.64, 1.01). Additionally, the policy slightly decreased the risk of preterm birth (OR = 0.94; 95% CI: 0.87, 1.03), while it did not affect GA. Effect estimates for extremely preterm and neonatal mortality were imprecise and inconclusive.

**Conclusions:**

This quasi-experimental and population based-study of 166,709 live births between 2013 and 2015 in Switzerland provides evidence of a reduction in the risk of LBW, VLBW and preterm birth thanks to a health policy that fully covered healthcare services during maternity.

**Key messages:**

• Free access to healthcare during pregnancy may mitigate adverse newborn health outcomes.

• A Swiss health policy that fully covered healthcare services during pregnancy reduced the risk of low birth weight and preterm births.

